# Enhancing quantum phase transitions in the critical point of Extended TC-Dicke model via Stark effect

**DOI:** 10.1038/s41598-018-29902-9

**Published:** 2018-08-02

**Authors:** Ahmed Salah, A. S. Abdel-Rady, Abdel-Nasser A. Osman, Samia. S. A. Hassan

**Affiliations:** 1 Mathematics and Theoretical Physics Department, Nuclear Research Center (NRC), Atomic Energy Authority, Cairo, 13759 Egypt; 2 Mathematics Department, Faculty of Science, South Valley University, Qena, Egypt; 3 Abdus Salam International Centre for Theoretical Physics (ICTP), Strada Costiera, 11 I - 34151, Trieste, Italy

## Abstract

A system of *N* two-level atoms, Tavis-Cummings Dicke (TC-Dicke) model, interacting with a one-mode electromagnetic radiation field in the presence of the Stark shifts is studied, which is expected to predict new phenomena that are not explored in the original TC-Dicke model. We obtained the potential energy surface of the system using a trial state the direct product of coherent states in each subspace. In the frame of mean-field approaches, the variational energy is evaluated as the expectation value of the Hamiltonian for this state. The order of the quantum phase transitions is determined explicitly and numerically. We estimate the ground-state energy and the macroscopic excitations in the superradiant phase. Moreover, we investigated the critical properties of the TC-Dicke model in the classical spin limit and coherent state. We observed that in the thermodynamic limit, the energy surface takes a simple form a direct description of the phase transition. Moreover, it is found that when the microwave amplitude changes the new phase transition occurs with the Stark shift. The analytical solutions and numerical results, which appear in this paper are agreement with our paper which published recently in Int. J. Mod. Phys. B when we studied the same model using a different coherent state.

## Introduction

Over the past decade, the ultra-cold atomic gases in optical cavities have presented as attractive new systems for studying strongly-interacting quantum many-body theories. It gives an opportunities to simulate quantum optical and condensed matter phenomena. The Hamiltonian Dicke model^1^ is one of the fundamental importance and the simplest models in the quantum optics, which describe the systems including the interaction between several atoms and an electromagnetic mode of radiation. This describes a large number of two-level atoms interacting with a single mode cavity field in the dipole approximation; the atoms are confined in a small cavity compared to the radiation wavelength. The Tavis-Cummings model^2^, which describes the interaction between a collection of N two-level atoms and a quantized field in the dipolar after performing a rotating-wave approximation has been used an extensive in quantum optics. Also, the small coupling strength near the resonant regime has been investigated. Quantum phase transition (QPT) or second-order phase transition play a very important role in many fields of modern physics such as condensed state physics, nuclear physics. The second-order QPT occurs at zero temperature when all thermal fluctuations are frozen out whereas quantum fluctuations are dominative. The phase transition in a simple form using the Glauber’s coherent states has been derived by Wang and Hioe^3^, who they calculated the free energy of the Dicke model, in the thermodynamic limit. Furthermore, the superradiant phase transition for molecules in the Dicke model has been proposed by Heep and Lieb^4^. Perturbation methods have been proposed to perform thermodynamical calculations without to have recourse to the Wang and Hioe computational method. Phase transitions have been intensely discussed for cavity QED systems and are known as superradiant phase transitions (SPTs) which are possible in the closely related circuit QED systems with capacitive coupling^5^. This means that the No-go theorem for superradiant quantum phase transitions in cavity QED does not apply and challenges the well-established analogy of circuit and cavity QED. So that a perturbative expansion of partition function and a simple analytic solution is found for high coupling constant has been developed^6^. Furthermore, the thermodynamic properties of the Dicke Hamiltonian have been treated^7^, in the framework of an approximate model, through a perturbative expansion of partition function. The Dicke QPT have been presented from the normal to the superradiant phase corresponds to the self-organization of atoms from the homogeneous into a periodically patterned distribution^8^. The Hamiltonian Dicke quantum-optical model describing the interaction of N two-level atoms with a number of bosonic modes have been demonstrated^9^. By means of Holstein-Primakoff transformation, a second-order phase transition for the Dicke model with the dipole-dipole interaction between the atoms has been revealed^10^. Furthermore, Viehmann, *et al*.^11^ claim that a superradiant phase transition for a chain of Cooper pair boxes capacitively coupled to a transmission line resonator is prevented by the Thomas-Reiche-Kuhn (TRK) sum rule for the electron dipole operator. Moreover, the equilibrium behavior of a superconducting circuit QED system containing a large number of artificial atoms has been investigated.

Despite the numerous publications on the extended Dicke model to investigate the QPT, the existence of this phase transition has been widely discussed. If the two-level atoms in the Dicke model correspond to atoms in a ground and excited state, and the transition is direct, then quantum mechanics forbids the transition.The extended Dicke models have been proposed to reveal rich phase diagrams^12,13^. furthermore, a spin squeezing, an intrinsic quantum property, in the Dicke model without the rotating-wave approximation have been studied and the distribution of the mean spin directions when quantum dynamics takes place have been investigated^14^. The dynamics of N coupled cavities, each containing an ensemble of N-identical two-level atoms in the slandered Dicke model have been discussed^15^. The generalized DM is used to study a gas of ultracold two-level atoms confined in a cavity, taking account of atomic center-of-mass motion and cavity-mode variations, and it is used to analyze separately the cases of a Gaussian, and a standing wave mode shape^16^. Also, Jaako, *et al*.^17^ have studied effective light-matter interactions in a circuit QED system from the standard Dicke model. Fortunately, the strong collective atom-field coupling in a coupling with an ultrahigh-finesse cavity filed has realized experimentally^18,19^. Recently, the Dicke quantum phase transition in an open system was experimentally observed self-organization of a BEC inside an ultrahigh-finesse optical cavity and pumped from a direction transverse to the cavity axis^20,21^. In the classical limit, the Dicke model undergoes an equilibrium phase transitions the coupling between the atoms and bosonic field reaches a specific value, which is considered one of the interesting phenomena. When increasing the collective coupling strength, this model exhibits a second-order quantum phase transition from a normal state to a superradiant state^22–24^. More recently, we study a system of a two-level atom BEC coupled to a high-finesse optical cavity interacting with a single mode electromagnetic field in the presence of the Stark-shift employing the energy surface method and the QPTs as well as Berry phase for this system is obtained^25^. Also, we obtain a simple expression for the energy surface of the Dicke Hamiltonian using as the Heisenberg-Weyl coherent states for the tensor product of matter and field components^26^.

The main contribution of this paper is a rigorous investigation for QPTs in a system of collective N two-level system interacting with a one-mode electromagnetic field. We review thoroughly the procedure to establish the Tavis-Cummings model in the regime of small but non zero frequency for a finite number N of atoms in the presence of Stark shifts. The reason for using Stark shifts is the shifting and splitting of spectral lines of atoms and molecules due to the presence of an external electric field.We obtained the good variational of ground energy state using the coherent states of U(2) to describe the matter part, together with the Heisenberg-Weyl coherent states for the description of the field. Furthermore, we obtained the energy surface for this model in the spin coherent state and the properties of the QPTs have investigated. We discuss how in the presence of Stark-shift modifies the results of the standard Tavis-Cummings Dicke model, which have been studied in our paper in Int. J. Theo. Phys^26^. We find that the QPTs are affected by the atom coupling strength and the Stark shift parameters. Compared with our work^25^, we notice that some of the properties for QPTs are changing under the consideration of a new coherent state.

## Results

### The effective Hamiltonian for the system

**Figure 1:**
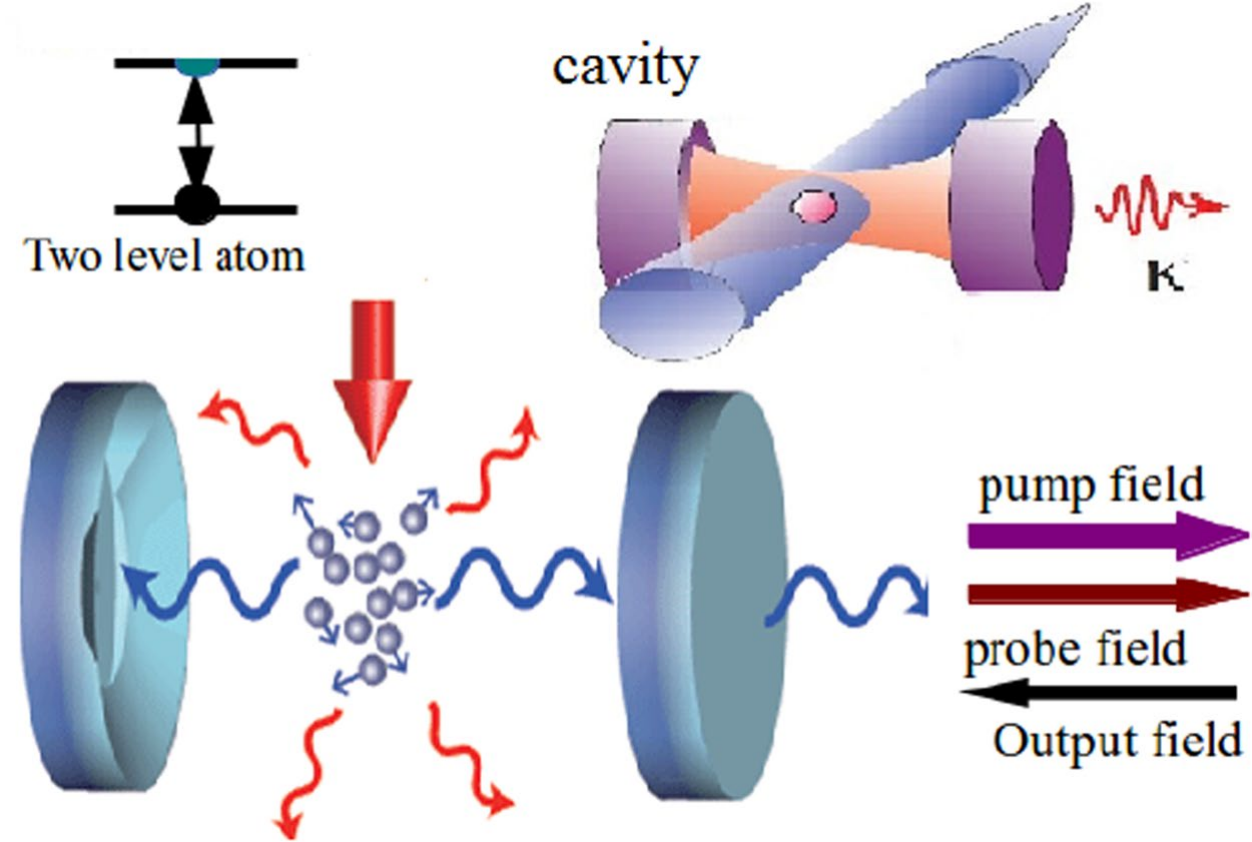
Schematic of the physical system under consideration: TC-Dicke model describing the interaction of an ensemble of two-level atoms in into a atomic BEC in a ultra high-finesse cavity with the Stark shifts.

In this section, we present the optomechanical system consists of an ultrahigh finesse single-mode optical cavity of frequency *ω* with N-identical two-level atoms with transition frequency *ω*_*a*_ are trapped in the quantized cavity shown schematically in Fig. 1. A high-finesse cavity provides good insulation against the environment. A good cavity can store photons for a long time before they are dissipated^24^. In the absence of the Stark-shift, the system under in our consideration is the same that investigated in the theoretical treatment^26^ and experimental observed^20^ $|{p}_{x},{p}_{y}\rangle =|0,0\rangle$ is mean the atom in ground state. Also, the scattering of photons between the pump and cavity field will couple *P* this ground state to a superposition state $|\hslash \overrightarrow{k},\hslash \overrightarrow{k}\rangle ={\sum }_{\mu ,\nu }|\mu \hslash \overrightarrow{k},\nu \hslash \overrightarrow{k}\rangle$. In this case, the single two-level atoms of mass *m* interacting with a single cavity mode and the standing-wave pump field. In a frame rotating with the pump laser frequency, the total Hamiltonian can be written as

1 $${\hat{H}}^{(1)}=\sum _{i=x,y}\,\frac{{P}_{i}^{2}}{2m}+{V}_{0}{\cos }^{2}\,ky+\delta ({\hat{a}}^{\dagger }+\hat{a})\,{\cos }^{2}kx\,{\cos }^{2}\,ky+({U}_{0}\,{\cos }^{2}\,kx-{{\rm{\Delta }}}_{c}){\hat{a}}^{\dagger }\hat{a}$$

where the first term is the kinetic energy of the atom with momentum operators *P*_*i*_ with *V*_0_ is a standing-wave potential of depth along the y-axis, *k* is the wave-vector of the light field, $\hat{a}$ and ${\hat{a}}^{\dagger }$ denote the annihilation and creation operators of the radiation field, respectively, which satisfy the bosonic commutation $[\hat{a},{\hat{a}}^{\dagger }]=1$, *U*_0_ is the light-shift of a single maximally coupled atom. The cavity resonance frequency *ω*_*c*_ is detuned from the pump laser frequency by Δ_*c*_. The Hamiltonian describing the consideration system is given by

2 $$\hat{H}={\hat{H}}_{I}+{\hat{H}}_{C}$$

The interaction Hamiltonian of BEC is given as

3 $${\hat{H}}_{I}=\int dxdy{\hat{{\rm{\Psi }}}}^{\dagger }(x,y){\hat{H}}^{(1)}\hat{{\rm{\Psi }}}(x,y)$$

The collision interaction Hamiltonian of BEC is given as

4 $${\hat{H}}_{C}=\int dxdy{\hat{{\rm{\Psi }}}}^{\dagger }(x,y){\hat{{\rm{\Psi }}}}^{\dagger }(x,y)\hat{{\rm{\Psi }}}(x,y)\hat{{\rm{\Psi }}}(x,y)$$

where $\hat{{\rm{\Psi }}}(x,y)$ denotes the atomic field operator, ${\hat{H}}^{(1)}$ is the single-particle Hamiltonian. The atomic field operator can be expanded in this basis, ${\rm{\Psi }}={\phi }_{1}{\hat{b}}_{1}+{\phi }_{2}{\hat{b}}_{2}$ where ${\hat{b}}_{1}$ and ${\hat{b}}_{2}$ are the bosonic operators. The number of atoms $\hat{N}={\hat{b}}_{1}^{\dagger }{\hat{b}}_{1}+{\hat{b}}_{2}^{\dagger }{\hat{b}}_{2}$ is assumed to be conserved by all processes. Inserting Ψ into the second quantized form, we can obtain the many-body Hamiltonian as follow^25^

5 $$\begin{array}{c}\hat{H}=\omega {\hat{a}}^{\dagger }\hat{a}+\frac{\chi }{4}\sum _{i,j=1}^{2}\,{(-1)}^{i+j}{\hat{b}}_{j}^{\dagger }{\hat{b}}_{j}{\hat{b}}_{i}^{\dagger }{\hat{b}}_{i}+\frac{{\omega }_{0}}{2}\sum _{j=1}^{2}\,{(-\mathrm{1)}}^{j+1}{{\hat{b}}_{j}}^{\dagger }{\hat{b}}_{j}\\ \,\,\,\,+\lambda \sum _{j < k}^{2}\,({\hat{a}}^{\dagger }+\hat{a})({\hat{b}}_{j}^{\dagger }{\hat{b}}_{k}+{\hat{b}}_{k}^{\dagger }{\hat{b}}_{j})+{\hat{a}}^{\dagger }\hat{a}\sum _{j=1}^{2}\,{\gamma }_{j}{{\hat{b}}_{j}}^{\dagger }{\hat{b}}_{j}\end{array}$$

where *ω* is the effective frequency of the cavity field^17^, *γ*_1_(*γ*_2_) is describe the intensity-dependent Stark-shift^25^, *χ* is the nonlinear coupling strength, which is satisfies, *χ* = *χ*_1_ = *χ*_2_ = −*χ*_12_, from equation (4), we obtain

6 $$\begin{array}{rcl}{\chi }_{1} & = & \frac{s}{2}\int {|{\phi }_{1}|}^{4}dxdy\\ {\chi }_{2} & = & \frac{s}{2}\int {|{\phi }_{2}|}^{4}dxdy\\ {\chi }_{12} & = & 2s\int {|{\phi }_{1}|}^{2}{|{\phi }_{2}|}^{2}dxdy\end{array}$$

For convenience, we use the Schwinger angular momentum operators, which is defined as

7 $$\begin{array}{c}{\hat{J}}_{z}=\frac{1}{2}({{\hat{b}}_{1}}^{\dagger }{\hat{b}}_{1}-{{\hat{b}}_{2}}^{\dagger }{\hat{b}}_{2}),\\ {\hat{J}}_{x}=\frac{1}{2}({{\hat{b}}_{1}}^{\dagger }{\hat{b}}_{2}+{{\hat{b}}_{2}}^{\dagger }{\hat{b}}_{1}),\\ {\hat{J}}_{y}=\frac{1}{2i}({{\hat{b}}_{1}}^{\dagger }{\hat{b}}_{2}-{{\hat{b}}_{2}}^{\dagger }{\hat{b}}_{1}),\\ {\hat{J}}_{\pm }={\hat{J}}_{x}\pm i{\hat{J}}_{y}\end{array}$$

In terms of the previous operators, we can rewrite the total Hamiltonian as^25^

8 $$\hat{H}=\omega {\hat{a}}^{\dagger }\hat{a}+\frac{1}{2}{\hat{a}}^{\dagger }\hat{a}({\gamma }_{1}+{\gamma }_{2})+{\hat{a}}^{\dagger }\hat{a}({\gamma }_{2}-{\gamma }_{1}){\hat{J}}_{z}+{\omega }_{0}\,{\hat{J}}_{z}+\chi \,{\hat{J}}_{z}^{2}+\lambda ({\hat{a}}^{\dagger }+\hat{a}){\hat{J}}_{x}.$$

The geometric interpretation of the Hamiltonian (5) can be derived using a trial state the direct product of coherent states in each subspace (Heisenberg-Weyl) coherent states. Therefore, the variational state constructed from the tensor product of matter and field components which can written in the form^26^

9 $$|\alpha ,\beta ,N\rangle ={e}^{{\tilde{\alpha }}^{2}\mathrm{/2}}{e}^{\tilde{\alpha }\hat{a}}\otimes \frac{1}{\sqrt{N!}}{({\hat{{\rm{\Gamma }}}}^{\dagger })}^{N}|0\rangle $$

where the $\hat{{\rm{\Gamma }}}$ operator is defined as

10 $$\hat{{\rm{\Gamma }}}=\frac{{\hat{b}}_{1}+\tilde{\beta }{\hat{b}}_{2}}{\sqrt{1+{\tilde{\beta }}^{2}}}$$

where *α* and *β* are the geometric parameters. $\tilde{\alpha }=\alpha {e}^{i\theta }$, $\tilde{\beta }=\beta {e}^{i\theta }$. Applying the corresponding coherent state representation of the Hamiltonian (5), the energy surface is

11 $$ {\mathcal E} (\alpha ,\beta ,\theta )=\omega {\alpha }^{2}+\frac{{\omega }_{0}}{2}\frac{{\beta }^{2}-1}{1+{\beta }^{2}}+\frac{\chi }{4}\frac{{\beta }^{4}-{\beta }^{2}+1}{{\mathrm{(1}+{\beta }^{2})}^{2}}+\frac{4\lambda \alpha \beta cos(\theta )}{1+{\beta }^{2}}+\frac{{\alpha }^{2}({\gamma }_{1}+{\gamma }_{2}{\beta }^{2})}{1+{\beta }^{2}}$$

**Figure 2:**
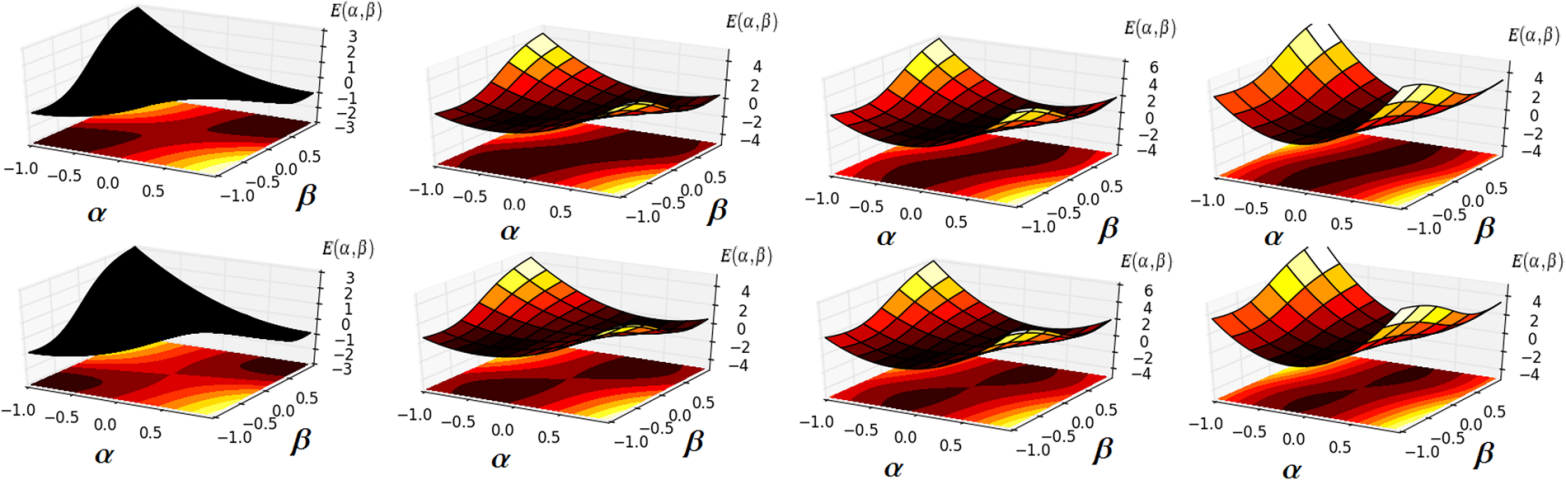
The contour plot and 3D plot of the scaled energy *E*(*α*, *β*) for *χ* = 0 (upper panels) and *χ* = 2 (lower panels). The black and orange regions indicate the regions with low and high energies, respectively. From left to right: for *γ*_1_ = 0.0 and *γ*_2_ = 0.0, *γ*_1_ = 1.0 and *γ*_2_ = 2.0, *γ*_1_ = 2.0 and *γ*_2_ = 4.0 and *γ*_1_ = 4.0 and *γ*_2_ = 5.0. Other parameters are unity.

In Fig. 2, we show the 3D and contour plots of the scaled energy. It can be seen that, either in the case of *χ* = 0 or in the case of *χ* = 2.0, the number of energy minimum changes from one to two in the (*α*, *β*)-plane with a different value of the Stark shifts parameter. We notice that during this translations the elliptic point becomes an unstable saddle point. This means that the QPTs are dramatically changed by increasing the Stark-shift parameters and strength coupling parameters.

The minimal for the energy surface is obtained *θ* = 0, *π*. It is not difficult to prove that a minimum is always obtained if the energy surface is rewritten as

12 $$E(\alpha ,\beta )=\omega {\alpha }^{2}+\frac{{\omega }_{0}}{2}\frac{{\beta }^{2}-1}{1+{\beta }^{2}}+\frac{\chi }{4}\frac{{\beta }^{4}-{\beta }^{2}+1}{{(1+{\beta }^{2})}^{2}}-\frac{4\lambda \alpha \beta }{1+{\beta }^{2}}+\frac{{\alpha }^{2}({\gamma }_{1}+{\gamma }_{2}{\beta }^{2})}{1+{\beta }^{2}}$$

where the displacements *α* and *β* should be determined from the equilibrium condition. After some derivations, we find that *α* is given by

13 $$\alpha =\frac{2\beta \lambda }{{\beta }^{2}{\gamma }_{2}+{\beta }^{2}\omega +{\gamma }_{1}+\omega }$$

Using Eq. (13), the scaled energy (12) as the function of *β* can be written as

14 $$E(\beta )=A{\beta }^{4}+B{\beta }^{2}+C$$

where

15 $$A=\frac{({\gamma }_{1}+\omega )(16{\lambda }^{2}+3\chi \omega -2\omega {\omega }_{0})+8{\gamma }_{2}{\lambda }^{2}+{\gamma }_{1}^{2}(3\chi -2{\omega }_{0})}{2{({\gamma }_{1}+\omega )}^{2}},$$

16 $$B=\frac{({\gamma }_{1}+\omega )(4{\omega }_{0}-3\chi )-16{\lambda }^{2}}{4({\gamma }_{1}+\omega )},$$

and

17 $$C=\frac{1}{4}(\chi -2{\omega }_{0})$$

Now, we analyse our result in equation (14). Firstly, the trivial constant term *C* can be neglected. The system is in a high-symmetry phase if and only if *λ* < *λ*_*c*_, i.e., *β* = 0. Furthermore, the system is in a low-symmetry phase when *λ* > *λ*_*c*_, there are two minima, i.e., $\beta =\pm \sqrt{-\,B\mathrm{/2}A}$. This means that symmetry breaking is associated with a second-order phase transition. The breaking of these symmetries are associated with rich quantum phases (It should be noticed that the wording “symmetry breaking” refers to “spontaneous symmetry breaking”, which is different from another nomenclature called “explicit symmetry breaking”)^23^. From Eqs (15 and 16), the nonzero collective excitation parameters can be obtained as follow

18 $${\beta }^{2}=\frac{({\gamma }_{1}+\omega )({\gamma }_{1}(3\chi -4{\omega }_{0})+16{\lambda }^{2}+3\chi \omega -4\omega {\omega }_{0})}{4({\gamma }_{1}(8{\lambda }^{2}+6\chi \omega -4\omega {\omega }_{0})+8{\gamma }_{2}{\lambda }^{2}+{\gamma }_{1}^{2}(3\chi -2{\omega }_{0})+\omega (16{\lambda }^{2}+3\chi \omega -2\omega {\omega }_{0}))}$$

Substituting equation (18) into equation (13), we have

19 $${\alpha }^{2}=\tfrac{16{\lambda }^{2}(3{\gamma }_{1}\chi -4{\omega }_{0}({\gamma }_{1}+\omega )+16{\lambda }^{2}+3\chi \omega )(8{\gamma }_{1}{\lambda }^{2}+8{\gamma }_{2}{\lambda }^{2}+6{\gamma }_{1}\chi \omega +3{\gamma }_{1}^{2}\chi -2{\omega }_{0}{({\gamma }_{1}+\omega )}^{2}+16{\lambda }^{2}\omega +3\chi {\omega }^{2})}{({\gamma }_{1}+\omega ){(32{\gamma }_{1}{\lambda }^{2}+48{\gamma }_{2}{\lambda }^{2}+27{\gamma }_{1}\chi \omega +3{\gamma }_{2}\chi \omega +12{\gamma }_{1}^{2}\chi +3{\gamma }_{1}{\gamma }_{2}\chi -4{\omega }_{0}({\gamma }_{1}+\omega )(2{\gamma }_{1}+{\gamma }_{2}+3\omega )+80{\lambda }^{2}\omega +15\chi {\omega }^{2})}^{2}}$$

**Figure 3:**
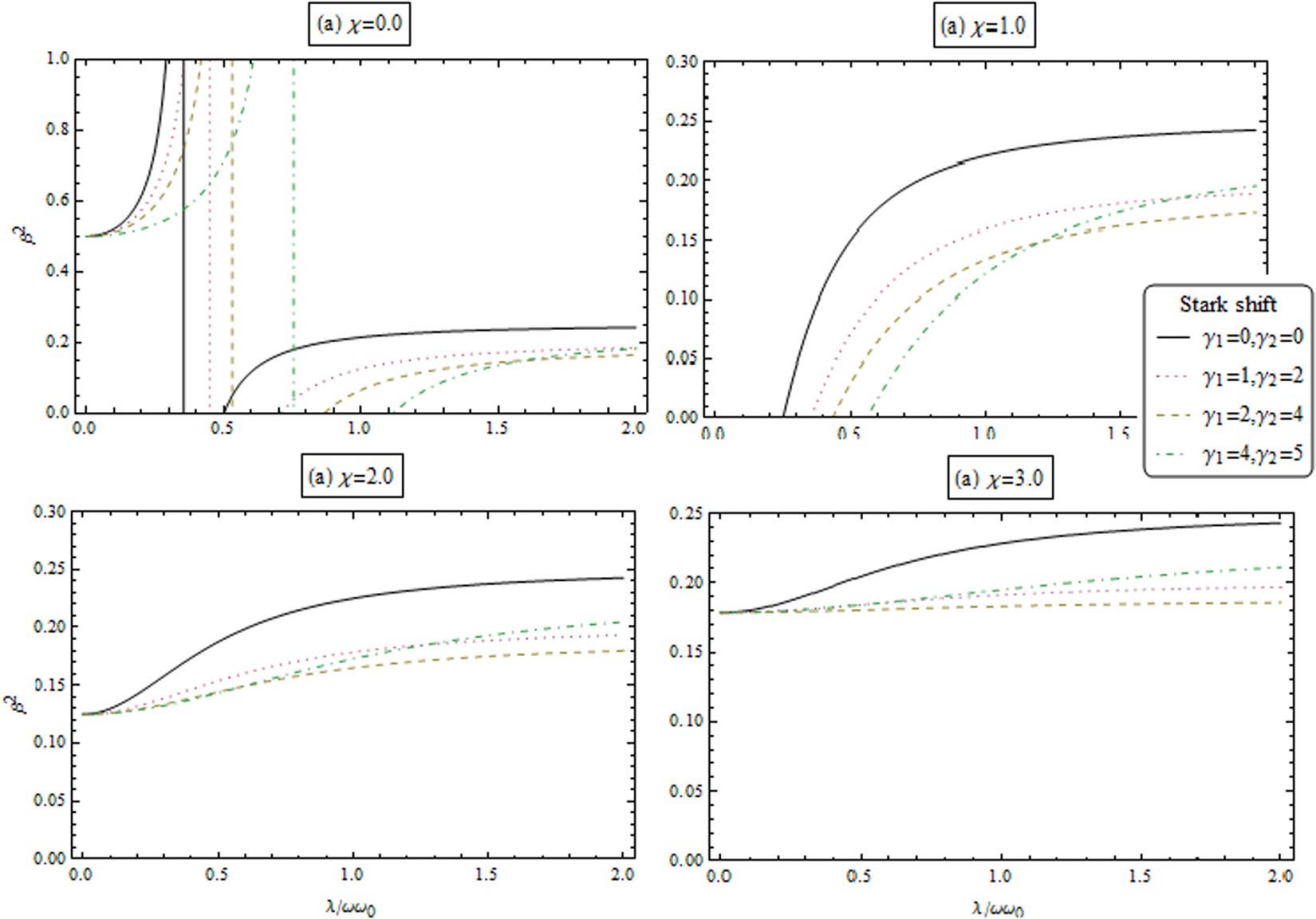
Macroscopic excitations for atom *β*^2^ as a function of coupling parameter *λ*/*ωω*_0_ for different coupling strength *χ* and Stark shift parameters.

**Figure 4:**
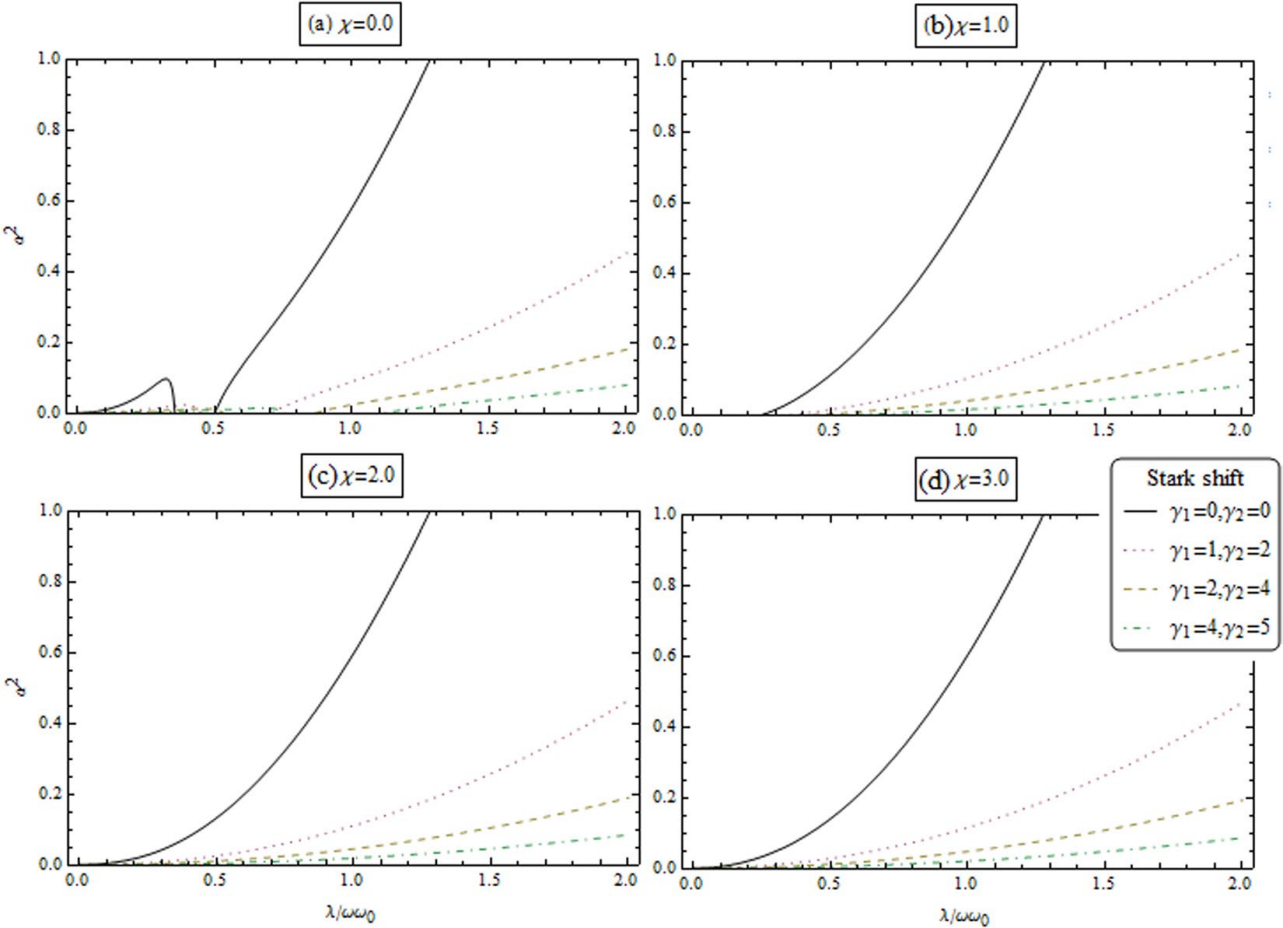
Macroscopic excitations for photon *α*^2^ as a function of coupling parameter *λ*/*ωω*_0_ for different coupling strength *χ* and Stark shift parameters.

Equations (18 and 19) describe the macroscopic quantum population of the collective excitations of the condensed atoms and the photon. Figures 3 and 4 illustrate the dependence of the macroscopic excitations for atoms (*β*^2^) and the photon (*α*^2^) as a function of *λ*/*ωω*_0_ for different value of coupling strength and the Stark shift parameters. From these figures, we remarked that the repulsive interactions (i.e., *χ* > 0) enhance the macroscopic excitations. Also, macroscopic excitations are enhanced by increasing the stark shift parameters.

The scaled ground-state of energy can be obtained from Eq. (14) after substituting Eq. (18) into Eq. (14), we get

20 $$\begin{array}{rcl}{E}_{0} & = & \frac{{\gamma }_{1}\chi (15{\gamma }_{1}\chi -32{\lambda }^{2}+30\chi \omega )+(16{\lambda }^{2}+3\chi \omega )(5\chi \omega -16{\lambda }^{2})}{32(8{\gamma }_{1}{\lambda }^{2}+8{\gamma }_{2}{\lambda }^{2}+6{\gamma }_{1}\chi \omega +3{\gamma }_{1}^{2}\chi -2{\omega }_{0}{({\gamma }_{1}+\omega )}^{2}+16{\lambda }^{2}\omega +3\chi {\omega }^{2})}\\  &  & +\frac{-\,8{\omega }_{0}(16{\gamma }_{2}{\lambda }^{2}+5{\gamma }_{1}\chi ({\gamma }_{1}+2\omega )+\omega (16{\lambda }^{2}+5\chi \omega ))+64{\gamma }_{2}{\lambda }^{2}\chi +16{\omega }_{0}^{2}{({\gamma }_{1}+\omega )}^{2}}{32(8{\gamma }_{1}{\lambda }^{2}+8{\gamma }_{2}{\lambda }^{2}+6{\gamma }_{1}\chi \omega +3{\gamma }_{1}^{2}\chi -2{\omega }_{0}{({\gamma }_{1}+\omega )}^{2}+16{\lambda }^{2}\omega +3\chi {\omega }^{2})}\end{array}$$

**Figure 5:**
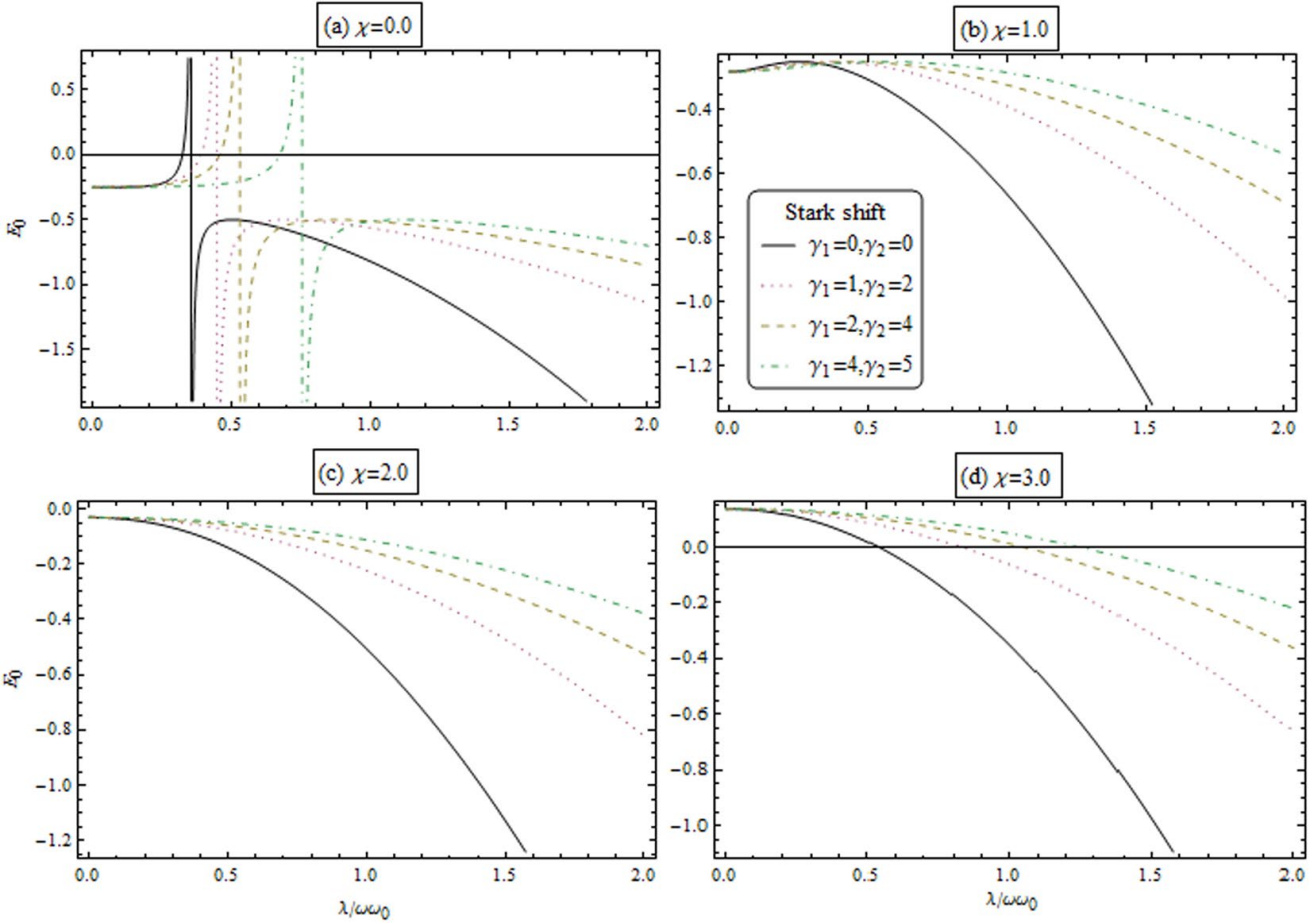
The scaled ground-state energy *E*_0_ as a function of BEC-cavity coupling parameter *λ*/*ωω*_0_ for different coupling strength *χ* and Stark shift parameters.

We show the changes of the ground-state energy by increasing the coupling strength in Fig. 5. For different values of the nonlinear parameter *χ* and the Stark-shifts, we found that the curves for the ground-state energy are continuous and smooth at the critical point. Furthermore, the system is essentially in the lower-energy state and is only microscopically excited in the normal phase.

### Mean-filed theory of QPT

In this section, we study the QPT in the spin coherent state with the mean-field approaches, the ground state wave function is given by

21 $$|\psi \rangle =|\alpha ,\zeta \rangle $$

where |*α*〉 and |*ζ*〉 are the boson and spin coherent states, respectively. The state of ground state energy is given by the expectation value of Hamiltonian (8)

22 $$\begin{array}{c}E(\alpha ,\theta )=\omega {\alpha }^{2}+\frac{1}{2}({\gamma }_{1}+{\gamma }_{2}){\alpha }^{2}-({\gamma }_{2}-{\gamma }_{1})j{\alpha }^{2}\,\cos \,\theta -{\omega }_{0}\,j\,\cos \,\theta \\ \,\,\,\,+\chi \,{j}^{2}{\cos }^{2}\theta +2j\lambda \alpha \,\sin \,\theta .\end{array}$$

By using the first equilibrium *∂E*(*α*, *θ*)/*∂α*, we get

23 $$\alpha =-\,\frac{2j\lambda \,\sin \,(\theta )}{{\gamma }_{2}({\omega }_{0}-2j\,\cos \,(\theta ))+{\gamma }_{1}(2j\,\cos \,(\theta )+{\omega }_{0})+2\omega }$$

Insert Eq. (23) in Eq. (22), the scale energy as a function *θ* can be written as

24 $$E(\theta )=-\,\frac{2{j}^{2}{\lambda }^{2}{\sin }^{2}(\theta )}{{\gamma }_{2}({\omega }_{0}-2j\,\cos \,(\theta ))+{\gamma }_{1}(2j\,\cos \,(\theta )+{\omega }_{0})+2\omega }+{j}^{2}\chi {\cos }^{2}(\theta )-j{\omega }_{0}\,\cos \,(\theta )$$

**Figure 6:**
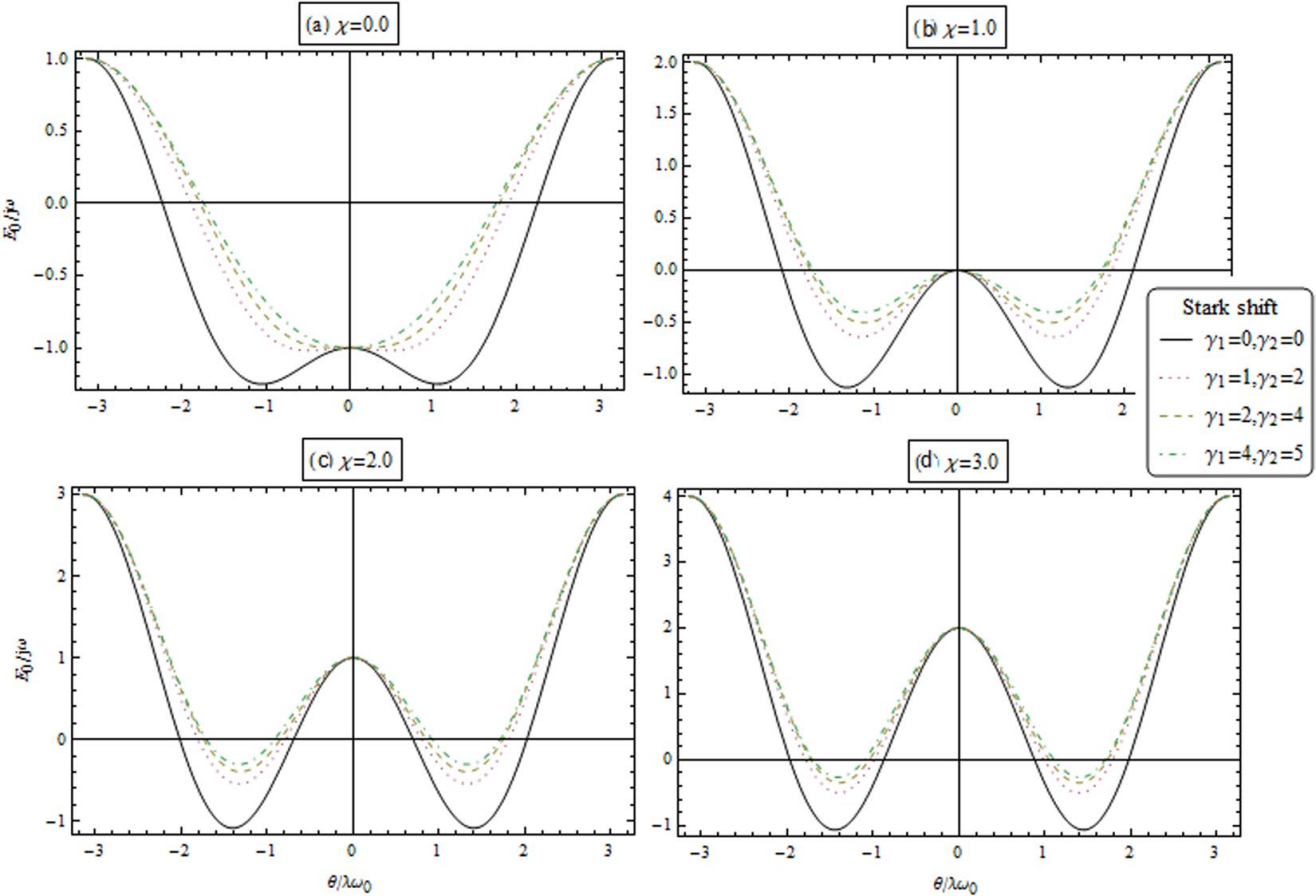
Energy functional *E*(*θ*)/*jω* versus *θ*/*λω*_0_ for different coupling strength *χ* and Stark shift parameters.

In Fig. 6, we plot the energy functional *E*(*θ*)/*jω* versus *θ*/*λω*_0_ for different coupling strength *χ* and Stark shift parameters. In Fig. 8, the contour plot of the scaled energy *E*(*α*, *θ*) for *χ* = 0 (upper panels) and *χ* = 2.0 (lower panels). From lift to right for *γ*_1_ = 0.0 and *γ*_2_ = 0.0, *γ*_1_ = 1.0 and *γ*_2_ = 2.0, *γ*_1_ = 2.0 and *γ*_2_ = 4.0 and *γ*_1_ = 4.0 and *γ*_1_ = 5.0. Other parameters are unity. The black and orange regions indicate the regions with low and high energies, respectively. During this transition, the stable point become unstable point. The energy function Eq. (24) is describe the second order phase transition, the minimal of energy function is satisfy the relation

25 $${A}_{1}\,\cos \,3\theta +{A}_{2}\,\cos \,2\theta +{A}_{3}\,\cos \,4\theta ={A}_{4}$$

where

$$\begin{array}{c}{A}_{1}=2{\gamma }_{1}^{2}{j}^{3}\chi +2{\gamma }_{2}^{2}{j}^{3}\chi -4{\gamma }_{1}{\gamma }_{2}{j}^{3}\chi ,\\ {A}_{2}=2({\gamma }_{1}-{\gamma }_{2}){j}^{2}({\gamma }_{1}\mathrm{(2}\chi -\mathrm{1)}{\omega }_{0}+{\gamma }_{2}\mathrm{(2}\chi +\mathrm{1)}{\omega }_{0}+{\lambda }^{2}+4\chi \omega ),\\ {A}_{3}=6{\gamma }_{1}^{2}{j}^{3}\chi +6{\gamma }_{2}^{2}{j}^{3}\chi -12{\gamma }_{1}{\gamma }_{2}{j}^{3}\chi +4{\gamma }_{1}j{\lambda }^{2}{\omega }_{0}-8{\gamma }_{1}j\omega {\omega }_{0}+8j{\lambda }^{2}\omega +8j\chi {\omega }^{2}\\ \,\,\,\,\,\,+4{\gamma }_{2}j{\lambda }^{2}{\omega }_{0}+2{\gamma }_{1}^{2}j\chi {\omega }_{0}^{2}+2{\gamma }_{2}^{2}j\chi {\omega }_{0}^{2}+4{\gamma }_{1}{\gamma }_{2}j\chi {\omega }_{0}^{2}+8{\gamma }_{1}j\chi \omega {\omega }_{0}+8{\gamma }_{2}j\chi \omega {\omega }_{0}\\ \,\,\,\,\,\,-4{\gamma }_{1}^{2}j{\omega }_{0}^{2}+4{\gamma }_{2}^{2}j{\omega }_{0}^{2}+8{\gamma }_{2}j\omega {\omega }_{0},\end{array}$$

and

$$\begin{array}{c}{A}_{4}=-\,{\gamma }_{1}^{2}{\omega }_{0}(2{j}^{2}\mathrm{(1}-2\chi )+{\omega }_{0}^{2})-{\gamma }_{2}^{2}{\omega }_{0}(2{j}^{2}\mathrm{(2}\chi +\mathrm{1)}+{\omega }_{0}^{2})-4{\omega }_{0}{\omega }^{2}\\ \,\,\,\,\,\,+{\gamma }_{1}({\gamma }_{2}(4{j}^{2}{\omega }_{0}-2{\omega }_{0}^{3})+6{j}^{2}{\lambda }^{2}+8{j}^{2}\chi \omega -4\omega {\omega }_{0}^{2})\\ \,\,\,\,\,\,-2{\gamma }_{2}({j}^{2}(3{\lambda }^{2}+4\chi \omega )+2\omega {\omega }_{0}^{2})\end{array}$$

The critical point *θ*_*c*_ when *χ* = *γ*_1_ = *γ*_2_ = 0 is given by^22^

26 $${\theta }_{c}=\arccos \,(\frac{\omega {\omega }_{0}}{2j{\lambda }^{2}})$$

**Figure 7:**
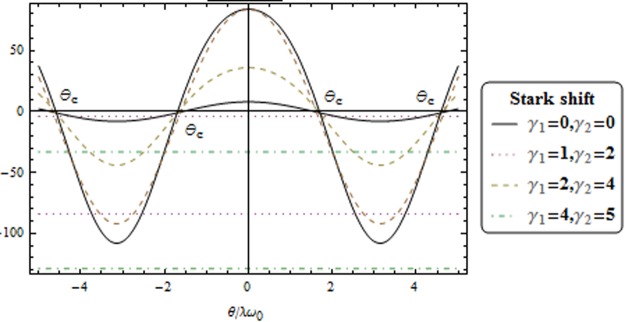
Graphical solutions of Eq. (25) for different values of Stark shift.

The solution of the Eq. (25) is *θ* = 0, Thus the system is in the normal phase. Also, the nonzero solution corresponds to the superradiant phase. In the case of *χ* ≠ 0 and for different Stark-shift parameters, the solution of Eq. (25) is not directly applicable to investigate the system. Now, we focus to study the behaviors of Eq. (25) and make some general statements about this solution. This will be done in Fig. 7. The left-hand side of Eq. (25) is a monotonically decreasing function for *θ* > 0 is local maximum and *θ* < 0 is a local minimum.

**Figure 8:**
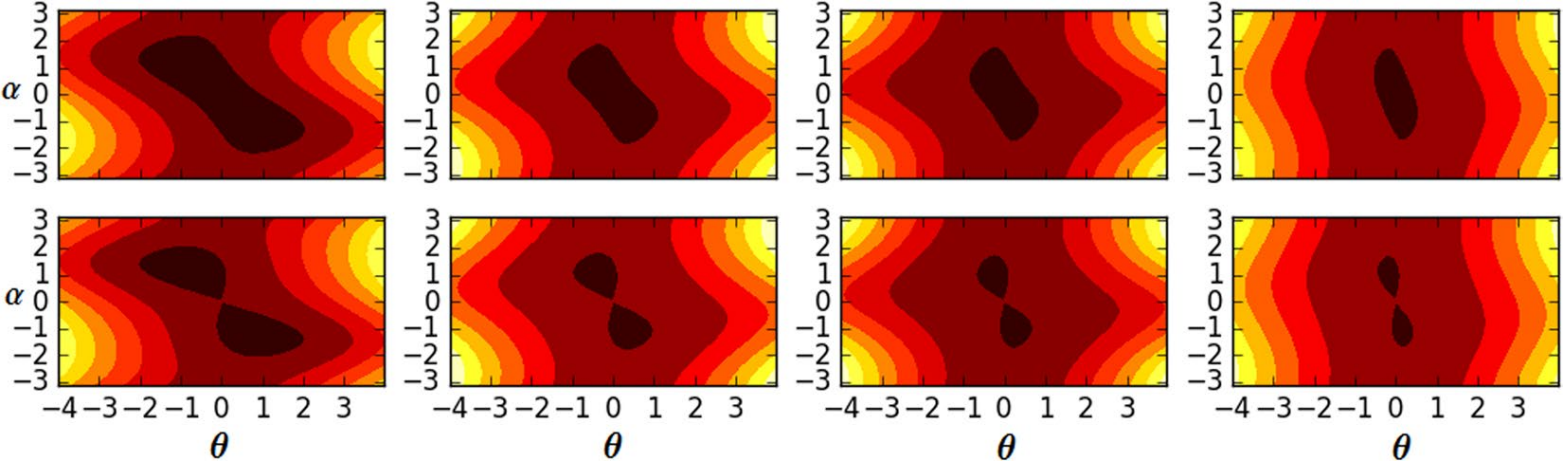
The contour plot of the scaled energy *E*(*α*, *θ*) for *χ* = 0 (upper panels) and *χ* = 1 (lower panels). The black and orange regions indicate the regions with low and high energies, respectively. From left to right: for *γ*_1_ = 0.0 and *γ*_2_ = 0.0, *γ*_1_ = 1.0 and *γ*_2_ = 2.0, *γ*_1_ = 2.0 and *γ*_2_ = 4.0 and *γ*_1_ = 4.0 and *γ*_2_ = 5.0. Other parameters are unity.

## Methods

### The determination of stable quantum phases

We first obtain the stable of quantum phases using the Hessian matrix in our case, we introduced the 2 × 2 Hessian matrix, the matrix elements can be calculated as

27 $$(\begin{array}{cc}\frac{{\partial }^{2}E(\alpha ,\beta )}{\partial {\alpha }^{2}} & \frac{{\partial }^{2}E(\alpha ,\beta )}{\partial \alpha \partial \beta }\\ \frac{{\partial }^{2}E(\alpha ,\beta )}{\partial \beta \partial \alpha } & \frac{{\partial }^{2}E(\alpha ,\beta )}{\partial {\beta }^{2}}\end{array})$$

If the Hessian matrix is positive definite (i.e., all eigenvalues of matrix are positive), quantum phases are stable, and vice versa

### Coherent and spin coherent states

The coherent state of the harmonic oscillation can be defined $\hat{a}|\alpha \rangle =\alpha |\alpha \rangle$, which can be written as

28 $$|\alpha \rangle ={e}^{\alpha {\hat{a}}^{\dagger }-{\alpha }^{\ast }\hat{a}}|0\rangle $$

The expectation values are given by

29 $$\begin{array}{c}\langle \alpha |{\hat{a}}^{\dagger }\hat{a}|\alpha \rangle ={|\alpha |}^{2}\\ \langle \alpha |{\hat{a}}^{\dagger }+\hat{a}|\alpha \rangle =2Re\alpha \end{array}$$

Furthermore, a spin coherent state^8^ is defined $|\zeta \rangle ={e}^{i\zeta {\hat{J}}_{y}}|j,-\,j\rangle$, which can be written as

30 $$|\zeta \rangle =\frac{1}{(1+{|\zeta |}^{2})}\sum _{m=-\,j}^{j}\,(\begin{array}{l}2j\\ \,j+m\end{array}){\zeta }^{j+m}|j,m\rangle $$

Expectation values of spin coherent states are given by

31 $$\begin{array}{c}\langle \zeta |{\hat{J}}_{x}|\zeta \rangle =j\,\sin \,\theta \\ \langle \zeta |{\hat{J}}_{z}|\zeta \rangle =-\,j\,\cos \,\theta \end{array}$$

## Discussion

In conclusion, we have studied the TC-Dicke model in the presence of the Stark-shift parameters and the rigorous investigation of the QPTs properties have been explored. We have obtained the variational energy surface of the TC- Dicke Hamiltonian using as the Heisenberg-Weyl coherent states for the tensor product of matter and field components. A very simple treatment has introduced to describe a QPTs present in TC- Dicke model for superradiance. We observed that the TC-Dicke QPT with Stark shifts can happen in an arbitrary coupling regime of the cavity field while the QPT in the standard Dicke model occurs only in the strong coupling regime of the cavity field and atoms. Also, the anti-resonant terms would bring a neglectful influence near the critical point and lead to the modification of the result of the QPTs. Furthermore, we remarked that the Stark-shift parameters can dramatically change quantum properties of the macro-quantum system. It is interesting to note that the interactions between the coherent atoms shift both the phase transition point and the macroscopic excitations in the superradiant phase. According to the current theoretical work and the recent experimental developments, we believe that it is possible to observe experimentally QPT with Stark-shift parameters by measuring the atomic population of the cavity field. Also, these Stark-shift parameters can also influence the entanglement and quantum Berry phase in the quantum information and quantum computing.
